# Emergency stomas; should non-colorectal surgeons be doing it? 

**Published:** 2018

**Authors:** Adnan Qureshi, Joanne Cunningham, Anil Hemandas

**Affiliations:** 1 *Northampton General Hospital NHS trust, UK*; 2 *Department of Cellular Pathology, Milton Keynes University Hospital NHS trust, UK*; 3 *Milton Keynes University Hospital, NHS trust, UK*

**Keywords:** Stoma, Colostomy, Non-colorectal surgeons, Hartman’s operation.

## Abstract

**Aim:**

The aim of this study was to compare general and stoma specific short term complications in patients having stoma surgery in either an emergency or elective setting during their index hospital stay. It also compares the complications specific to a stoma carried out by surgeons with or without a specialist interest in colorectal surgery.

**Background:**

The stoma created in emergency surgery has a high short and long term complication rate. Emergency stomas where the site has not been marked preoperatively by a stoma therapist are more prone to complications. These complications may severely affect a patient’s quality of life.

**Methods:**

We retrospectively analysed data for all non-urological stomas created over the last three years in our institute. This covered the period from January 2014 to January 2017. The stoma care department kept a full database record of all patients. Besides demography we analysed the type of stoma i.e. colostomy or ileostomy, indications for the stoma, most common operation, length of stay (LOS) and short term complications based on the Clavien-Dindo classification. We also analysed the perioperative stoma related complications within the emergency cohort.

**Results:**

A total of 199 patients had new ostomies created during the three-year period. Four patients died during the inpatient stay and were excluded from the analysis. The total number of stomas created in the emergency cohort was 60 and 135 stomas were elective procedures. The male to female ratio was 1:1.01. The average age for the emergency cohort was 6 years older than for the elective cohort. There was a statistically significant difference in length of stay between the two cohorts (T Test P Value =.02). There was a higher number of elective patients discharged in the first week compared to the emergency surgery patients. The rate of grade 3 or 4 complications was higher in the emergency cohort of patients. The rate of grade 3 or 4 complications was also much higher in patients operated by surgeons who did not have a specialist interest in colorectal surgery. The majority of grade 3 complications seen in the emergency surgery cohort and operated on by non-colorectal specialists (NCS) were stoma related, i.e retraction, necrosis and prolapse.

**Conclusion:**

Emergency surgery procedures are frequently bowel related. Emergency stoma surgery should not be taken as trivial procedure, non-colorectal surgeons should take advice and assistance from specialist colorectal surgeons for bowel related cases, particularly when a stoma is involved

## Introduction

 The first deliberate surgical creation of a stoma was in the early 18th century when a stoma was created to divert faecal material in anal agenesis. The high perioperative mortality rate subsequently discouraged most surgeons from carrying out this procedure. Following the development of anaesthesia during the mid-1800s, stoma surgery became a realistic treatment option and surgeons in the mid to late 1800s used diverting colostomy to manage bowel obstruction. The first recorded local complications were reported in the 1820’s. However, it was not until the twentieth century, towards the end of the World War One when this became a widespread surgical technique. A successful technique for ileostomy was described by Brooks in 1952 and this is still in use today. A stoma can be permanent or temporary and with the success of improved perioperative care, stomas are now routinely made during emergency and elective surgery (1,2).

In the elective setting trends are more towards an ileostomy as a temporary stoma, for example, defunctioning in a low anterior resection and colostomy as a permanent stoma which is easier to manage. The role of a defunctioning stoma for low anterior resection is well established, although defunctioning procedures do not decrease the anastomotic leak rate but does affect the consequences of small anastomotic leak in relation to the rate of a second operation (3). For elective patients preoperative planning and counselling are extremely important prior to the creation of an acceptable and functional ostomy. Even with the improved stoma care support and counselling in elective settings, the psychological effects of having a stoma can affect the short and long term outcomes.

In emergency surgery a stoma may be a lifesaving procedure to control sepsis during the acute illness, this normally get reversed when the patient has fully recovered. Due to time constraints pre-operative counselling and planning are not possible especially when operating out of hours. In the emergency setting stoma formation is often regarded as the least important part of an operation, which may be relegated to the most junior member of the operating team.

The stoma created in emergency surgery has a high short and long term complication rate. Emergency stomas where the site has not been marked preoperatively by a stoma therapist are more prone to complications. These complications may severely affect a patient’s quality of life (4).

We have retrospectively evaluated 199 operations where a stoma was created. These were carried out at a single centre over a three-year period. We analysed the data and identified the short term local and systemic complications related to both emergency and elective cohorts. 

## Methods

This was a retrospective analysis of data for all of the ostomy patients over the past three years covering the period from January 2013 to January 2016, using the electronic patient record system. The department of surgery has five full time colorectal surgeons (CS), and five general surgeons with no colorectal interest (NCS) who all participate in the emergency on-call rota. The department stoma care team keep a detailed record of all the patients who have had a stoma.

The inclusion criteria for the study were: 

(1) All patients who had a non-urological stoma created in the three-year period.

(2) Patient age >16 years.

The exclusion criteria was any patient who died within 48 hours of admission.

There were four patients excluded from the analysis who died in early post-operative period i.e with in 48hours of surgery due to causes not related to stoma surgery directly. We included all remaining patients who had either emergency surgery or an elective stoma during this period. Patients were divided into two cohorts of elective and emergency surgery. The patient’s demography, indication for the stoma, type of stoma (i.e. colostomy or ileostomy, loop or end), intension of the stoma (i.e. temporary or permanent), length of hospital stay (LOS) and early complications, were all analysed. We also reviewed whether the stoma had been reversed. 

We identified the number of stoma procedures carried out by the CS and NCS and compared the complication rates between the two cohorts. We used the Clavien-Dindo classification to compare the short term complication rate during the inpatient hospital stay. We also recorded how many patients given a temporary stoma subsequently their stomas had reversed. The long term complications were not included in this study. 

## Results

**Figure 1:**
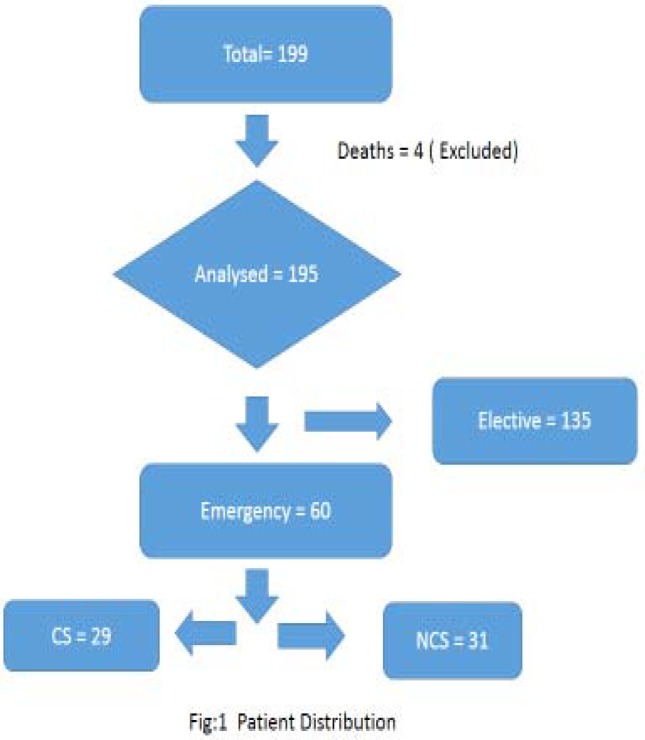
Patients Distribution

199 patients were identified over the three-year study period. Four patients died within forty eight hours of their hospital admission and were therefore excluded from the analysis. Out of these four deaths only one was after elective surgery due to massive MI and three after emergency stoma for ischemic bowel. None of the deaths were directly related with stoma surgery. The patients distribution is shown in Figure 1.

The mean age was 63± 21 years in the emergency cohort and 57± 19 years in the elective cohort. 

**Figure 2:**
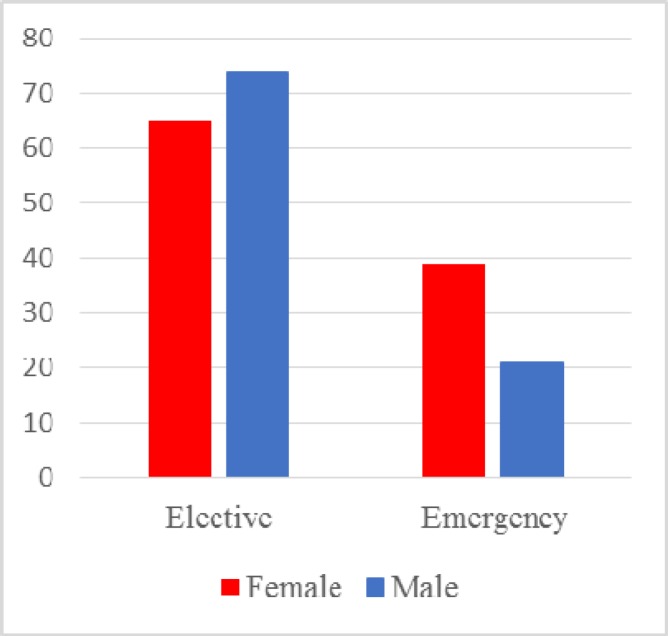
Male; Female ratio

There were 102 female and 93 male patients. The male to female ratio in the elective and emergency surgery cohorts is shown in figure 2. Most emergency stomas were female patients compared to male patients, who had predominantly elective stomas.

**Figure 3:**
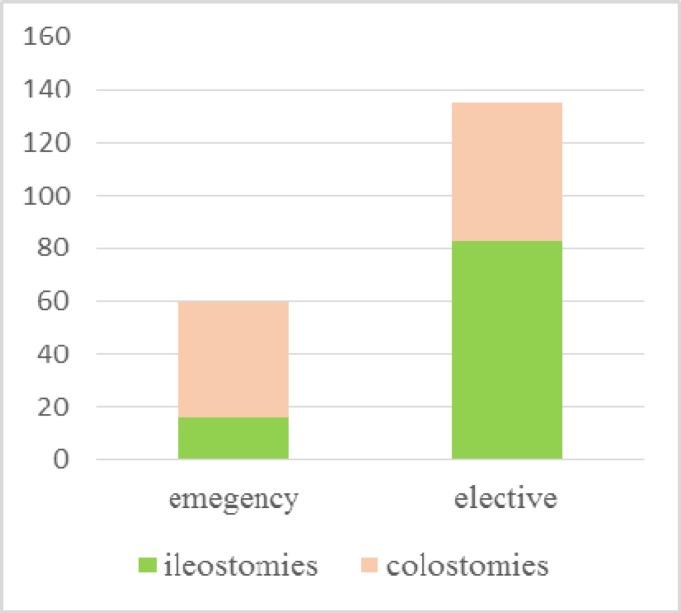
Type of Stoma: Emergency Vs Elective n=199

Figure 3 shows the type of stomas made in the emergency and elective cohorts, predominant stomas in the elective setting were ileostomy as compared to colostomy in the emergency cohort. Surprisingly, only 60 stomas were created during emergency surgery, with the most common type of stoma made being an end colostomy (Hartman’s procedure). The emergency indications for stoma formation are shown in table 1. The most common indication was perforated diverticular disease followed by large bowel obstruction secondary to colorectal cancer. Two colostomies were required secondary to iatrogenic trauma during gynaecological surgery. Overall, 62% (37 cases) of emergency stomas were later reversed. Patients with major co-morbidities and being high risk for a second operation were the most common reason for non-reversal of the stoma. 

**Table 1:** Diagnosis and type of stoma in the emergency cohort (n=60)

Diagnosis	Ileostomy n=16	Colostomy n=44
Diverticular Perforation	0	17
Colorectal Adenocarcinoma	4	12
Volvulus	2	6
Inflammatory bowel disease	2	0
Ischemia	6	3
Trauma	2	2
Stercoral perforation.	0	4

**Table 2:** Diagnosis and type of stoma in the elective cohort (n=135)

Diagnosis	Ileostomy n=83	Colostomy n=52
Colorectal adenocarcinoma	65	29
Diverticular Disease	2	18
Ischemic Stricture	1	1
IBD	5	2
Constipation	10	2

**Figure 4:**
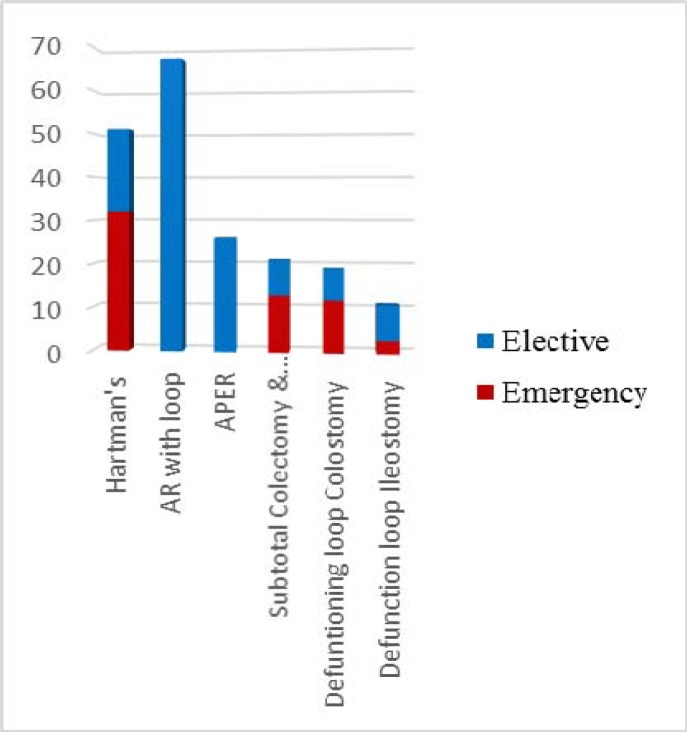
Type of operations: Emergency Vs Elective

There were 135 elective stomas undertaken. Table 2 shows diagnosis and indication for stoma surgery in the elective cohort while figure 4 shows the type of operations in both the elective and emergency cohorts. 

**Figure 5:**
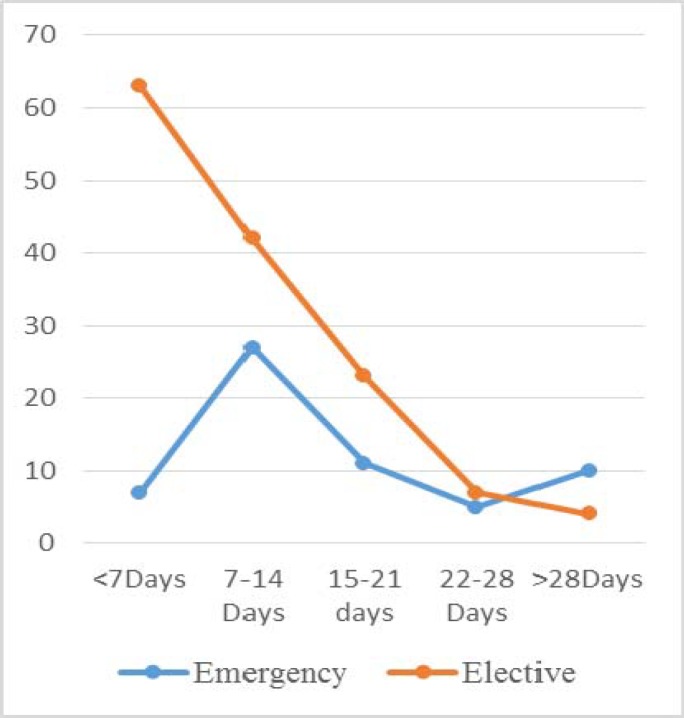
Length of Stay: Elective Vs Emergency

The most common stoma type was an ileostomy and the most common indication was defunctioning after rectal surgery. 77% (103 cases) of the ileostomy cohort were later reversed. The main reason for non-reversal in the elective group was patient choice and this was followed by patient comorbidities. There were 52 colostomies made in the elective setting including low Hartman’s, APER and defunctioning colostomies. 

The average length of stay was higher in the emergency group then in the elective group. The median stay was 11 days in the elective cohort and 14 days in the emergency cohort. (T-Test P Value 0.02). Figure 5 shows 46% of elective patients and 11% of emergency patients were discharged home within one week of surgery. In the second week post-surgery the majority (45%) of patients discharged were in the emergency group. After the second week there was no difference between the two cohorts. 

There were three deaths in the emergency surgery cohort. The causes for which were multi-factorial including pulmonary embolism, hospital acquired pneumonia and myocardial infarction. These patients were generally unwell and had a metabolic abnormality on admission. They were operated on for a life threatening condition and the reason for the stomas was because they could not tolerate an anastomosis or lengthy procedure. This explains the high mortality rate in this group. There was one death in the elective cohort, this was due to a massive myocardial infarction in HDU in the early post-operative period.

The complication rate was graded using the Clavien-Dindo classification (see appendix 1). There is no significant difference in grade 1 and grade 2 complications in emergency and elective stoma patients. However, there is a significantly higher rate of grade 3 and grade 4 complications in the emergency cohort compared to the elective cohort. This is shown in table 3.

**Appendix 1:**
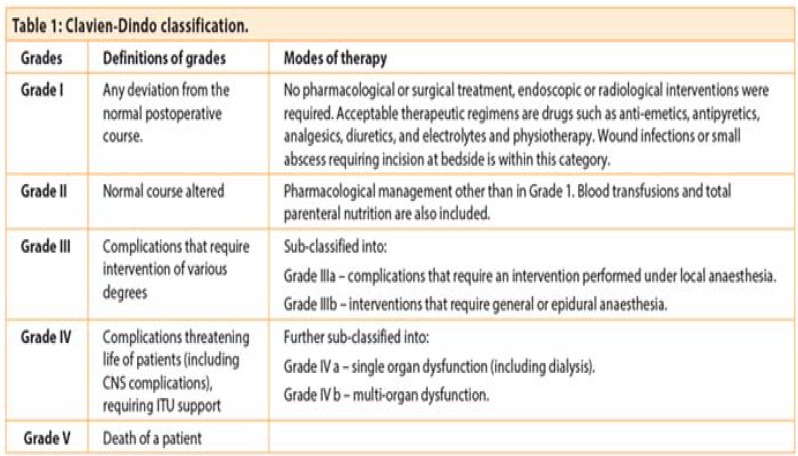
Clavien-Dindo classification

**Figure 6:**
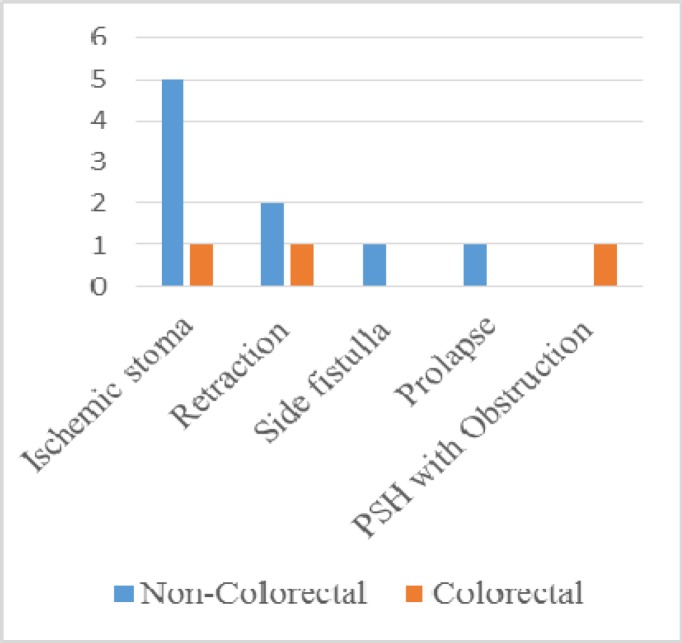
Stoma Complications (n=60)

All emergency surgeries are directly supervised by consultant surgeons. Almost half of the patients (51%, 31 cases) from the emergency cohort were operated on by NCS. The emergency cohort showed that the rate of grade 3 and grade 4 complications was much higher in the patients operated on by NCS. The majority of complications in this group were related to the stoma (see figure 6). This includes ischemic stoma, retraction and side fistula, all requiring a second operation.

**Table 3:** Complication rates in emergency verses elective stoma surgery

	Elective n=135	Emergency n=60
Grade I	14 (10%)	5 (8%)
Grade 2	33 (24%)	19 (31%)
Grade 3	08 (6%)	12 (20%)
Grade 4	2 (1%)	5 (8%)

## Discussion

On review of the literature several studies were found to show that patients undergoing emergency colorectal surgery are normally older than those having elective surgery (3). This is mainly due to the effective screening programme and increased awareness in the United Kingdom which is decreasing colonic cancer mortality. Our study also showed this trend, with the mean age in the emergency cohort being 6 years older than the elective group. 

The literature does not show any gender specific difference for emergency colorectal surgery. However, elective colorectal surgery has a slightly higher incidence toward male patients (5). This was also true in our study which showed a slight male predominance in the elective group. Whilst the emergency surgery group showed a slight female predominance. 

Colonic emergencies remain major life threatening conditions associated with high morbidity and mortality rates (6). Non-specific abdominal symptoms account for their delayed presentation especially in elderly patients with multiple co-morbidities and limited metabolic reserve. In our study there is a significant difference in the mortality rate among the emergency and elective groups. The mortality rate in the emergency cohort was related to the underlying pathology and was not found to be a direct complication of the surgery. 

It is relatively common to create a stoma in both emergency and elective bowel surgery. They are necessary for several colonic and rectal conditions and represent a major change in patient quality of life. Previous studies have discussed risk factors, complications and outcomes of stomas made in both the emergency and elective setting (7). 

Emergency stomas are shown to have a higher complication rate (8). This was also true in our study which showed a higher rate of complications in the emergency cohort. We found the majority of complications were grade 1 and 2. However, surprisingly there was a significant difference for grade 3 complications between the two groups which were directly related to the stoma and lead to a second operation. 

The choice of stoma in emergency surgery is disease depend. In our study most of the stomas were end colostomy secondary to a recto-sigmoid resection (Hartmann’s procedure). As compared to elective settings it is the surgeons’ choice to bring an ileostomy or a colostomy for defunctioning after an anastomosis (9). Technically emergency stomas are much more difficult to create due to oedema and inflammation of the bowel. Experience in elective colorectal surgery can certainly improve the outcomes in these settings.

It has been discussed that single stage colonic surgery with primary anastomosis in selected cases is deemed safe, but mainly in specialist hands. In many studies the optimal strategy proposed for perforated diverticulitis was a resection with a primary anastomosis with defunctioning stoma (10) and the colorectal surgeons were more likely to perform a single stage operation. 

The impact of surgical specialisation in emergency colorectal surgery shows significant differences in outcome when operated by colorectal surgeons (8). In many regions (e.g. United States, Australasia) colorectal surgery exists as a separate surgical specialty (11). However, in Europe colorectal surgery is not yet a formally accepted specialty. Similarly, with the introduction of emergency surgical speciality jobs in the United Kingdom the concept of specialist management in complex colonic diseases is dying. Most of the cases were performed by emergency surgeons, where in fact, a decision and an experienced colorectal surgical hand can improve the overall outcome. This is especially true in cases that require a stoma.

Stoma creation is not a trivial undertaking. It involves a careful surgical technique to minimise the complication rate (which may be relatively frequent) and promotes good ostomy function. Various studies have reported stoma complication rates of 21–70 % (12). 

Approximately a fifth of stomas have to be sited in emergencies. These stomas may be left permanently due to patient choice or co-morbidities preventing further surgery, which was also shown in our study. Therefore, selecting the site for stoma placement is important for a good outcome. This has been discussed in the literature which shows in the absence of input for marking and placement of the stoma, from a stoma specialist nurse or surgeon with colorectal interest, the outcomes are poor (13). 

Studies suggest that specialist colorectal surgeons should manage colorectal emergencies for a better patient outcome (4). Patients with colonic obstruction due to colonic cancer or perforated diverticulitis may present in a critical condition. They require an experienced colorectal surgeon who can make the best surgical management plan for the patient. A surgeon with colorectal experience can confidently deal with any intraoperative decisions in what may be complex bowel surgery. This has been shown to achieve the best patient outcomes (14-16).

As previous studies have shown clear outcome differences between general and colorectal surgeons for bowel related operations, our study has focused on the stoma and the related short term outcomes. This is very important with regards to patient morbidity and in some cases can influence long term outcomes such as the patient’s quality of life. 

We acknowledge that this study is subject to the limitations of a single centre retrospective study. Nevertheless, our data is a useful guide for future efforts attempting to improve outcomes in emergency colorectal surgery, in particular surgery that involves creating a stoma. We also highlight the need for input from a colorectal surgeon for the best patient outcome. It may be difficult to control many disease related factors. Although in emergencies colorectal specific cases like stoma formation can have a better outcome in the presence of a colorectal surgeon. We believe that as a consequence of this study, more through audits can be done to convince health authorities to organise emergency procedures in such a way that speciality based trained surgeons are in charge of handling their respective emergencies
